# Seasonality of Coastal Picophytoplankton Growth, Nutrient Limitation, and Biomass Contribution

**DOI:** 10.3389/fmicb.2021.786590

**Published:** 2021-12-06

**Authors:** Javier Alegria Zufia, Hanna Farnelid, Catherine Legrand

**Affiliations:** ^1^Marine Phytoplankton Ecology and Applications Laboratory (MPEA), Department of Biology and Environmental Science, Centre for Ecology and Evolution in Microbial Model Systems (EEMiS), Linnaeus University, Kalmar, Sweden; ^2^School of Business, Innovation and Sustainability, Halmstad University, Halmstad, Sweden

**Keywords:** *Synechococcus*, picoeukaryotes, phycoerythrin, phycocyanin, Baltic Sea, nitrate, ammonium, temperature

## Abstract

Picophytoplankton in the Baltic Sea includes the simplest unicellular cyanoprokaryotes (*Synechococcus*/*Cyanobium*) and photosynthetic picoeukaryotes (PPE). Picophytoplankton are thought to be a key component of the phytoplankton community, but their seasonal dynamics and relationships with nutrients and temperature are largely unknown. We monitored pico- and larger phytoplankton at a coastal site in Kalmar Sound (K-Station) weekly during 2018. Among the cyanoprokaryotes, phycoerythrin-rich picocyanobacteria (PE-rich) dominated in spring and summer while phycocyanin-rich picocyanobacteria (PC-rich) dominated during autumn. PE-rich and PC-rich abundances peaked during summer (1.1 × 10^5^ and 2.0 × 10^5^ cells mL^–1^) while PPE reached highest abundances in spring (1.1 × 10^5^ cells mL^–1^). PPE was the main contributor to the total phytoplankton biomass (up to 73%). To assess nutrient limitation, bioassays with combinations of nitrogen (NO_3_ or NH_4_) and phosphorus additions were performed. PE-rich and PC-rich growth was mainly limited by nitrogen, with a preference for NH_4_ at >15°C. The three groups had distinct seasonal dynamics and different temperature ranges: 10°C and 17–19°C for PE-rich, 13–16°C for PC-rich and 11–15°C for PPE. We conclude that picophytoplankton contribute significantly to the carbon cycle in the coastal Baltic Sea and underscore the importance of investigating populations to assess the consequences of the combination of high temperature and NH_4_ in a future climate.

## Introduction

Marine picophytoplankton (here defined as autotrophic cells <2 μm in diameter) is a diverse group consisting of picocyanobacteria (*Prochlorococcus*, *Synechococcus*, and *Cyanobium*), and photosynthetic picoeukaryotes (PPE). In oligotrophic systems, they account for more than 50% of the total chlorophyll a (Chl *a*) (Peña et al., 1990; Agawin et al., 2000; Durand et al., 2001) and can be responsible for most of the primary production (Magazzu and Decembrini, 1995; Uitz et al., 2010; Rii et al., 2016). Their success in low-nutrient environments is mainly explained by their small size, which provides them with a high surface-to-volume ratio, resulting in a competitive advantage for nutrient uptake compared to larger phytoplankton cells (Partensky et al., 1999; Pittera et al., 2014). Picophytoplankton are also abundant in eutrophic waters (Caroppo, 2015), in both coastal (Calvo-Díaz et al., 2004; Morán, 2007) and estuarine systems (Paerl et al., 2020; Sathicq et al., 2020), suggesting that they have a significant role in carbon cycling and ecosystem functions. Understanding which factors control picophytoplankton biomass is important for determining their role in the marine food web.

In the Baltic Sea, an estuary-like semi-enclosed large brackish water body, picophytoplankton are a key component of the phytoplankton community and reports of their contribution to Chl *a*, as a proxy for biomass, ranges from 15 to 85% (Sondergaard et al., 1991; Stal et al., 1999, 2003; Ohlendieck et al., 2000; Tamm et al., 2018). Baltic Sea picophytoplankton consists of the simplest cyanoprokaryotes *Synechococcus* and *Cyanobium* (hereafter referred to as picocyanobacteria; Flombaum et al., 2013; Celepli et al., 2017) and PPE. *Cyanobium* is a genera closely related to *Synechococcus* which has typically been associated with freshwater (Komárek et al., 1999; Callieri and Stockner, 2002; Sánchez-Baracaldo et al., 2005). The large contribution and diversity of picocyanobacteria in the Baltic Sea have recently been recognized (Stal et al., 2003; Herlemann et al., 2011; Bertos-Fortis et al., 2016; Hu et al., 2016; Celepli et al., 2017), but there is currently no systematic monitoring of picocyanobacteria in the Baltic Sea Proper. Coastal areas in the Baltic Sea are dynamic, and nutrient runoff has been related to increase in picocyanobacteria abundances (Lagus et al., 2007); however, abundance measurements in coastal areas remain scarce. Likewise, the PPE community is thought to be diverse and important in coastal ecosystems, but their distribution and abundance in the Baltic Sea are largely unknown (Kuosa, 1991; Tamm et al., 2018). Although knowledge of the picophytoplankton in the Baltic Sea is increasing, information about the ecological role, community composition, and distribution of picophytoplankton, especially in coastal areas, is lacking.

Picocyanobacteria can be divided into two populations based on the presence of the two phycobiliprotein pigments phycoerythrin (PE), and phycocyanin (PC) (Stomp et al., 2007; Liu et al., 2014; Tamm et al., 2018). PE-rich picocyanobacteria (PE-rich) strains are adapted to blue and green light absorption, and phycocyanin-rich picocyanobacteria (PC-rich) strains are adapted to red light absorption. Consequently, PE-rich are better adapted to low-turbidity waters (generally open waters); meanwhile, PC-rich are better adapted to turbid waters (generally coastal waters) (Campbell and Carpenter, 1987; Vörös et al., 1998; Stomp et al., 2007; Rajaneesh et al., 2015). The Baltic Sea picocyanobacteria community is dominated by a unique PE pigment cluster (Mazur-Marzec et al., 2013; Larsson et al., 2014; Tamm et al., 2018). However, PC-rich populations have been observed to have similar contributions to the picocyanobacterial community as PE-rich in areas with higher turbidity such as the Gulf of Finland, and some coastal areas (Stomp et al., 2007; Haverkamp et al., 2008). PE-rich and PC-rich frequently co-occur in estuarine environments, and recent studies from diverse coastal areas suggest that physio-ecological adaptations to nutrients and temperature affect their distribution and seasonality (Rajaneesh et al., 2015; Jiang et al., 2016; Paerl et al., 2020).

Picophytoplankton seasonal dynamics are determined by temperature, light, nutrient concentration (Otero-Ferrer et al., 2018; Hunter-Cevera et al., 2019; Fowler et al., 2020), and biotic factors (Ohlendieck et al., 2000; Ploug et al., 2011). The few studies focusing on picophytoplankton seasonality in the Baltic Sea suggest that their community composition and abundance have a strong seasonal variation (Kuosa, 1991; Bertos-Fortis et al., 2016). Maximum picocyanobacteria cell concentrations of 10^5^–10^6^ cells mL^–1^ have been observed during summer, coinciding with high-temperature and low-nutrient conditions (Kuosa, 1991; Albertano et al., 1997; Hajdu et al., 2007; Tamm et al., 2018). Peak cell abundances for PPE of up to 10^3^ cells mL^–1^ have been reported during the autumn in a non-stratified water column (Kuosa, 1991). Thus, the dynamics and relative importance of picocyanobacteria and PPE along different seasons are expected to vary. In a global perspective, PPE appear to be better adapted to low temperatures and NO_3_ availability while picocyanobacteria is generally favored by high temperatures and NH_4_ availability (Glibert et al., 2016; Otero-Ferrer et al., 2018). Picocyanobacteria preference of NH_4_ over NO_3_ has been observed in laboratory experiments on isolates (Moore et al., 2002; Tan et al., 2019). Similar observations from natural populations have also been reported from bulk populations in nutrient addition bioassays during summer phytoplankton blooms (Stal et al., 1999; Kuuppo et al., 2003; Lagus et al., 2007). This was also confirmed by recent studies at the single-cell level (Berthelot et al., 2018; Klawonn et al., 2019). In the Baltic Sea, the occurrence of picocyanobacteria during late summer has been linked to the presence of dinitrogen (N_2_)-fixing cyanobacterial blooms (Ohlendieck et al., 2000; Stal and Walsby, 2000; Ploug et al., 2011), suggesting that interactions with larger phytoplankton may be an important factor driving seasonal dynamics of picophytoplankton. However, disentangling the nutrient effect from other factors such as temperature is necessary to understand the seasonal changes in picophytoplankton community composition.

This study aims to explore the abundance, apparent net growth rate, and nutrient limitation of three populations of picophytoplankton, i.e., PE-rich, PC-rich, and PPE, over an annual cycle at a coastal sampling station located in the Baltic Sea Proper. Weekly field sampling was conducted in March to December in 2018, and a series of nutrient addition bioassays were performed to assess how nutrient limitation drives picophytoplankton growth. This study provides high-resolution data on the dynamics of picophytoplankton and nutrient controls during different seasons. The results show that the response and contribution to total biomass of the different populations vary with nutrients and season and highlight the importance of picophytoplankton to the total phytoplankton community throughout the year.

## Materials and Methods

### Field Sampling

The sampling was carried out at the K-station (56°39′25.4″N 16°21′36.6″E, 3 m deep, Supplementary Figure 1), a station located in the southeast coast of Sweden, in the Kalmar Sound, in the city of Kalmar. The Kalmar Sound waters are eutrophic and are highly influenced by coastal anthropogenic activities, particularly agriculture. This has affected the yearly primary production, which has roughly doubled over the last century (Legrand et al., 2015). Surface water (1 m depth) was sampled weekly using a Ruttner sampler and a 10-L acid-washed polycarbonate carboy. Sampling lasted from March until December 2018, covering spring (March, April, May), summer (June, July, August), autumn (September, October, November), and winter (December). Temperature and salinity were measured using a conductivity/temperature/depth sensor (CTD^®^ Castaway). Water from the carboy bottle was filtered through a 200-μm mesh gauze to remove large particles. Samples were collected for dissolved inorganic nutrients, Chl *a*, and pico- and larger phytoplankton abundance measurements. Water for the nutrient addition bioassays was collected on 11 occasions. Experiments were initiated within 1 h of sample collection and incubated in the laboratory under controlled conditions for 48 h.

### Abiotic and Biotic Parameters

Samples for dissolved inorganic nutrients (NO_2_ + NO_3_ and PO_4_, SiO_2_, TN, and TP) were filtered (400 mL) through a GF/F filter and frozen at −20°C until analysis using standard protocols (UV-Spectrophotometer, Valderrama, 1995). Chl *a* was extracted and measured following Jespersen and Christoffersen (1987). Briefly, 50–200 mL seawater was filtered on (A/E) glass fiber filters in duplicates (∼1 μm pore size, Pall Life Sciences, Ann Arbor, MI, United States) under low vacuum and extracted in ethanol (96%) in darkness. Chl *a* concentrations were measured using a Turner fluorometer (Turner design Model #040, Tucson, AZ, United States). Samples for larger phytoplankton (>5 μm) community composition were collected and counted microscopically (Nikon TMS, Tokyo, Japan) after preservation with acidic Lugol’s solution (1% final concentration) following Utermöhl (1958). Phytoplankton was identified to genus or species level, and cell measurements were used to calculate the carbon biomass following Edler (1979); Olenina et al. (2006), and HELCOM Phytoplankton Expert Group (2013).

Samples for picophytoplankton abundance were fixed with glutaraldehyde solution Grade I 25% in H_2_O (Sigma-Aldrich, MO, United States; 1% final concentration) and stored at −80°C until flow-cytometry analysis. Cells of PE-rich and PC-rich and PPE were identified and counted using a CyFlow^®^ Cube 8 flow cytometer (Partec^®^, Jettingen-Scheppach, Germany) equipped with a blue pumped solid-state laser (20 min W) at 488 nm and a red laser diode (25 mW) at 638 nm. For each sample, 50 μL was analyzed at an average flow rate of (10 μL s^–1^). For the cell characterization, four optical parameters were used at a logarithmic scale: forward scatter (FSC) as a proxy for cell diameter, FL2 (590/50 nm, blue laser dependent) as a proxy for PE content, FL3 (675/50 nm, blue laser dependent) as a proxy for Chl *a*, and FL4 (675/50 nm, red laser dependent) as a proxy for PC content.

Picocyanobacteria were identified and separated into two groups depending on their specific pigment characteristics: PE-rich with a high FL2 signal and PC-rich with a high FL4 signal. PPE was identified based on larger diameter and higher FL3 signal compared to the other groups. The diameters were estimated in the FSC with the help of 1- and 3-μm beads. For a more detailed description of the picophytoplankton identification, see Supplementary Information and Supplementary Figure 2. Gating and visualization of the flow cytometric data were carried out using the R (version 3.6.1) packages flowCore, flowWorkspace, openCyto, and CytoRSuite (Hammill, 2019). The relative contribution of picophytoplankton based on the carbon biomass concentration was estimated for each value included in Supplementary Table 1.

### Nutrient Addition Bioassays at *in situ* Temperature

To examine the seasonal variation of nutrient (nitrogen and phosphorus) limitation of picophytoplankton at the K-station, a total of 11 short-term (48 h) nutrient addition bioassays were conducted during different seasons (see Supplementary Table 2): in spring (7th and 22nd of May), summer (4th and 21st of June, 3rd of July and 29th of August), autumn (12th and 25th of September and 9th and 23rd of October), and winter (11th of December). The bioassay treatments were nutrient addition nutrients in excess concentrations: NH_4_ (200 μM), NO_3_ (200 μM), PO_4_ (10 μM), NO_3_ (200 μM) + PO_4_ (10 μM), NH_4_ (200 μM) + PO_4_ (10 μM), and controls without nutrient addition (Supplementary Figure 3). Triplicates were done for each treatment in 650-mL acid-washed polycarbonate bottles. The bioassays were incubated in the laboratory at a light intensity of 90 μE m^–2^ s^–1^, a photoperiod of 12L:12D, at *in situ* temperature provided with a constant inflow and outflow of seawater. Bottles were shaken manually every 24 h to avoid sedimentation. The abundance of PE-rich, PC-rich, and PPE were measured at the beginning and end of each experiment. The apparent net growth rate was calculated according to:

$$\mathrm{Apparent}\,\mathrm{net}\,\mathrm{growth}\,\mathrm{rate}\,\left({\mathrm{day}}^{-1}\right)=\frac{\ln\left(N_{T\,48}/N_{T\,0}\right)}{2\,\mathrm{days}}$$

Where N refers to the cell concentration, T48 refers to the last day of the experiment, and T0 refers to the day of the experimental setup.

### Statistical Analysis

A principle component analysis was used to analyze the relationship between the picophytoplankton cell abundance and measured biotic and abiotic variables: PE-rich, PC-rich, PPE, NO_2_ + NO_3_, PO_4_, SiO_2_, temperature, salinity, phytoplankton biomass (total biomass, dinoflagellates, diatoms, total cyanobacteria, and N_2_-fixers) and Chl *a*. All variables were transformed [log(x + 1)] prior the analysis.

The effect of nutrient limitation in the bioassays was analyzed by comparing the apparent net growth rates under different nutrient conditions. Each bioassay was analyzed separately using one-way ANOVA (*n* = 18, *p* < 0.05) followed by Tukey’s range test (*p* < 0.05) to test the statistical differences between treatments. Normality and heteroscedasticity were assessed *via* quantile–quantile plots and Cochran’s *C* test (*p* = 0.05), respectively. All statistical analyses were performed using R version 3.6.1 (R Core Team, 2019).

## Results

### Field Observations

**FIGURE 1:**
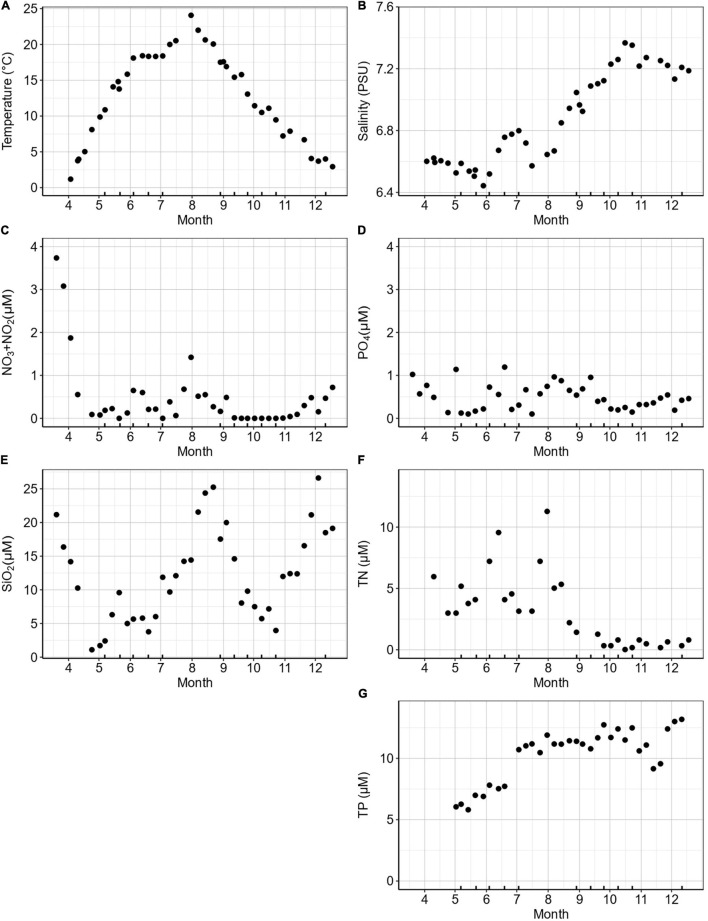
K-station weekly measurements during 2018 for **(A)** temperature (°C), **(B)** salinity (PSU), **(C)** NO_2_ + NO_3_ (μM), **(D)** PO_4_ (μM), **(E)** SiO_2_ (μM), **(F)** total N (TN; μM), and **(G)** total P (TP; μM). Ticks on the *x*-axis mark the dates when a bioassay was performed.

At the K-station, seawater temperature (1 m depth) ranged from 0 to 24°C from early spring to mid-summer and 18–3°C from autumn to winter (Figure 1A). Temperature was above 20°C from July to September. Salinity varied from 6.5 PSU during spring–summer after the ice melting period up to 7.5 PSU in October after a dry summer (Figure 1B). The highest concentrations of dissolved inorganic nitrate (NO_2_ + NO_3_) were recorded in mid-March (4.3 μM; Figure 1C). Nitrate concentrations decreased gradually to the end of the spring and remained low throughout the summer and autumn (<0.06–0.7 μM) followed by a small rise in the winter. Concentrations of NH_4_ were not recorded for 2018 but typically ranged between 1 and 2.52 μM at the K-station during 2019 and 2020 (data not shown). The concentrations of dissolved inorganic phosphorus (PO_4_) decreased in spring (1 to 0.2 μM) with occasional peaks of up to 1.2 μM between March and September (Figure 1D). Silicate (SiO_2_) concentrations ranged from 1.1 to 26.6 μM with strong peaks in summer, autumn, and early winter (Figure 1E). Total nitrogen (TN) levels ranged from 1.4 to 11.2 μM during spring–summer and decreased to below detection during autumn–winter (Figure 1F). Total phosphorus (TP) levels increased from spring (5.8 μM) to winter (14.1 μM; Figure 1G).

### Phytoplankton Dynamics

**FIGURE 2:**
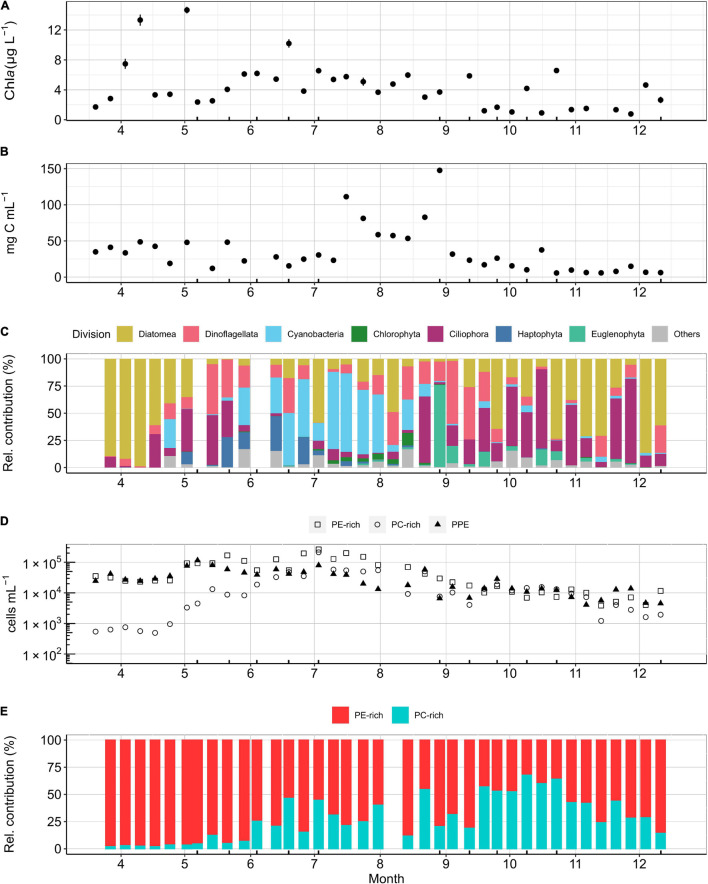
K-station weekly measurements for **(A)** Chl *a* (μg L^– 1^), **(B)** total phytoplankton (>5 μm in diameter) carbon biomass concentration (mg C m^– 3^) based on microscopy, **(C)** relative contribution of phytoplankton divisions (>5 μm in diameter) to total biomass (mg C m^– 3^) calculated from microscopic counts microscopy, **(D)** PE-rich, PC-rich, and PPE cell concentration in a logarithmic scale (cells mL^– 1^), and **(E)** relative contribution of PE-rich and PC-rich (%) based on flow cytometry cell counts. Ticks on the *x*-axis mark the dates when a bioassay was performed.

Chl *a*, as a proxy for phytoplankton biomass, showed seasonal variation with a spring bloom maximum in April (up to 15 μg L^–1^), relatively constant summer concentrations (4–8 μg L^–1^), and a gradual decline after late autumn (<4 μg L^–1^; Figure 2A). In the spring, the phytoplankton community was dominated by diatoms reaching a maximum biomass of 52 mg C m^–3^ (Figures 2B,C). After the spring bloom, biomass dropped to 25–15 mg C m^–3^ consisting of a diverse community of dinoflagellates, haptophytes, ciliates, and large cyanobacteria (Figure 2C). During summer, as temperatures increased above 20°C, filamentous nitrogen (N_2_)-fixing cyanobacteria, dominated by *Aphanizomenon* (87% relative contribution in June), *Dolichospermum* (69% relative contribution in July), and *Nodularia spumigena* (91% relative contribution in August), bloomed with peaks up to 74.4 mg C m^–3^. In late summer, the larger phytoplankton community composition fluctuated, recording a maximum biomass of 148 mg C m^–3^ due to a bloom of Euglenophyta (75% relative contribution). During autumn, the phytoplankton community biomass decreased to a minimum of 6 mg C m^–3^ and was dominated by ciliates, dinoflagellates, and diatoms. The biomass remained low during early winter, and the phytoplankton community was dominated by diatoms (Figures 2B,C).

Cell abundances of picophytoplankton showed a strong seasonality that differed for the three groups (Figure 2D). PPE had highest abundance during May and PE-rich and PC-rich reached maximum cell abundances in July. PE-rich and PC-rich maximum cell abundances (PE-rich: 2.6 × 10^5^ cells mL^–1^, PC-rich: 2.1 × 10^5^ cells mL^–1^) were more than double of that of PPE (1.1 × 10^5^ cells mL^–1^). In spring, the picocyanobacteria community was strongly dominated by PE-rich cells (Figure 2E). During summer, PC-rich increased its cell abundance to 50% of the picocyanobacteria community. During autumn, PC-rich had a maximum cell abundance of 65% but decreased down to 10% by the end of November (Figure 2E).

**FIGURE 3:**
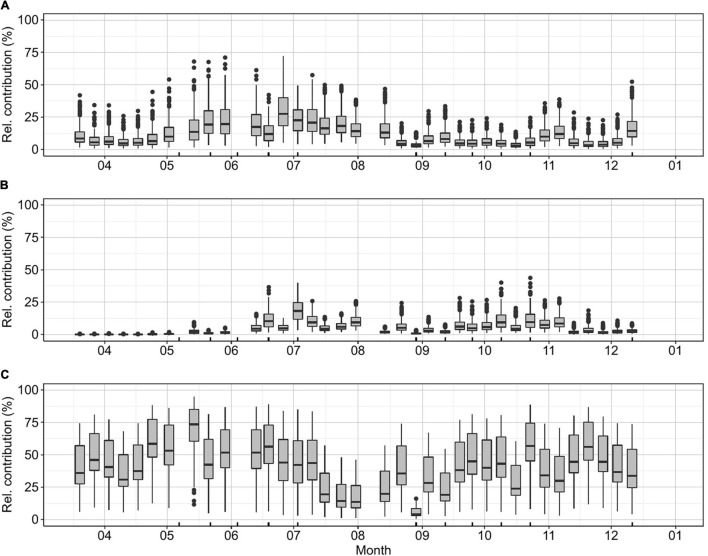
K-station weekly relative carbon biomass contribution to the total phytoplankton community for **(A)** PE-rich, **(B)** PC-rich, and **(C)** PPE. The relative contribution of picophytoplankton based on the carbon biomass concentration was estimated for each value included in Supplementary Table 1. Ticks on the *x*-axis mark the dates when a bioassay was performed.

Literature values of picophytoplankton carbon biomass conversion factors show a large variation depending on the methods used for determination, the season, region, or taxon in the case of PPE (Supplementary Figure 4 and Supplementary Table 1). In this study, we estimated the median, minimum, and maximum possible contributions of picophytoplankton to the total phytoplankton community (Figure 3). Picophytoplankton median contribution was 53% on average throughout the sampling period. Picophytoplankton maximum contributions were recorded from May to early July (max. May 14th, median: 89% relative contribution). The highest contribution of PE-rich and PC-rich occurred during summer (Figure 3A, PE-rich June 26th, median: 27% relative contribution, Figure 3B, PC-rich July 3rd, median: 18% relative contribution). The PPE median contribution to the total carbon biomass was close to 40% for most of the year, with a maximum relative contribution of 73% during May. The minimum contribution of PPE took place in the period between mid-July and early August (median 3–19% relative contribution; Figure 3C).

**FIGURE 4:**
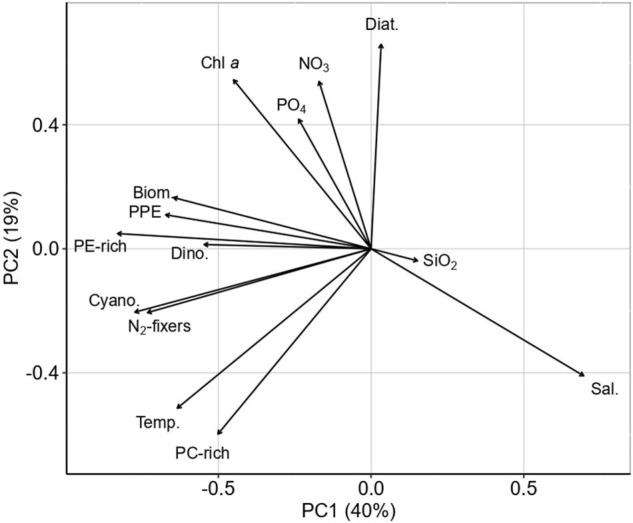
Principal component analysis ordination plot (PC1 × PC2) of PE-rich, PC-rich, PPE, NO_2_ + NO_3_, PO_4_, SiO_2_, temperature, salinity, phytoplankton biomass (total phytoplankton biomass (Biom.), dinoflagellates (Dino.), diatoms (Diat), total cyanobacteria (Cyano), and N_2_-fixers), and Chl *a*.

The principal component analysis explained 52% of the variation in the two first principal components (Figure 4). PC1 accounted for 35% of the variance with a negative load of PE-rich, PC-rich, PPE, NO_2_ + NO_3_, PO_4_, temperature, phytoplankton biomass (total biomass, dinoflagellates, total cyanobacteria, and N_2_ fixers), and Chl *a* (Fig. PCA). PC2 accounted for 17% of the total variation with a negative load of total PC-rich, cyanobacteria, N_2_-fixers, temperature, SiO_2_, and salinity.

### Nutrient Limitation of Picophytoplankton

Apparent net growth rates for PE-rich (−0.07 to 0.82 day^–1^), PC-rich (−1.10 to 0.78 day^–1^), and PPE (−0.01 to 0.91 day^–1^) were in line with previous observations in the Baltic Sea but were generally lower than rates reported from tropical and subtropical environments (Tables 1, 2).

**TABLE 1:** Bioassay average apparent net growth rates (day^–1^) on each date and treatment for PE-rich (day^–1^), PC-rich (day^–1^), and PPE (day^–1^) calculated from cell abundances measured using flow cytometry at T0 and T48.

DATE	Control	PO_4_	NO_3_	NO_3_ + PO_4_	NH_4_	NH_4_ + PO_4_	Effect
**PE-rich**							
2018-05-07	0.83	1.05	1.12	1.21	0.92	1.17	NO_3_↑, PO_4_↑
2018-05-22	0.34	0.27	0.41	0.27	0.70	0.47	NH_4_↑
2018-06-04	0.42	0.46	0.51	0.43	0.62	0.66	NH_4_↑
2018-06-19	0.38	0.29	0.21	0.31	0.40	0.44	NO_3_↓
2018-07-03	–0.01	0.04	0.01	0.01	0.34	0.36	NH_4_↑
2018-08-29	0.19	0.10	0.09	0.11	0.15	0.19	–
2018-09-12	–0.03	–0.11	–0.03	0.02	–0.08	0.04	–
2018-09-25	0.34	0.35	0.26	0.35	0.49	0.34	NH_4_↑
2018-10-09	–0.08	–0.03	0.10	–0.01	–0.02	0.08	–
2018-10-23	–0.03	0.07	0.10	0.04	0.00	–0.05	–
2018-12-11	–0.05	–0.09	–0.01	–0.01	–0.06	–0.01	–
**PC-rich**							
2018-05-07	–1.11	–1.28	–1.15	–0.94	–1.48	–1.39	–
2018-05-22	–0.53	–0.48	–0.57	–0.60	–0.53	–0.58	—
2018-06-04	–0.12	–0.05	–0.10	–0.08	0.00	0.06	NH4↑, PO4↑
2018-06-19	0.14	0.07	0.00	0.04	0.17	0.18	–
2018-07-03	–0.51	–0.49	–0.61	–0.53	–0.08	0.11	NH_4_↑
2018-08-29	0.43	0.36	0.40	0.40	0.39	0.32	–
2018-09-12	0.78	0.77	0.82	0.83	0.87	0.78	–
2018-09-25	0.34	0.45	0.38	0.40	0.42	0.42	–
2018-10-09	0.51	0.59	0.91	0.91	0.84	0.82	NH_4_↑, PO_4_↑
2018-10-23	0.40	0.58	0.61	0.56	0.52	0.60	NO_3_↑, PO_4_↑
2018-12-11	–0.10	–0.05	–0.06	–0.10	0.02	–0.02	–
**PPE**							
2018-05-07	0.52	0.61	0.53	0.52	0.57	0.55	PO_4_↑
2018-05-22	0.40	0.37	0.40	0.37	0.50	0.50	NH_4_↑
2018-06-04	0.33	0.31	0.17	0.30	0.33	0.34	NO_3_↓
2018-06-19	0.20	0.09	0.04	0.07	0.14	0.14	NO_3_↓, PO_4_↓
2018-07-03	–0.01	–0.03	0.17	0.21	0.20	0.32	NH_4_↑, PO_4_↑
2018-08-29	0.45	0.33	0.37	0.31	0.26	0.27	NH_4_↓
2018-09-12	0.56	0.57	0.57	0.54	0.57	0.47	–
2018-09-25	0.27	0.40	0.22	0.21	0.29	0.36	PO_4_↑
2018-10-09	0.91	0.81	0.80	0.96	0.90	0.89	–
2018-10-23	0.77	0.64	0.63	0.65	0.82	0.72	–
2018-12-11	0.47	0.43	0.40	0.36	0.48	0.47	–

*The column on the right shows the significant effect of the nutrients, indicating ↑ when the effect is positive and ↓ when the effect is negative.*

**TABLE 2:** Compilation of reported net growth rates (day^–1^) of *Synechococcus* sp. and PPE from incubation experiments.

Picocyanobacteria				
Area	Temperature (°C)	Dates	Net growth rate (day^–1^)	References
Baltic Sea (K-station)	6.8–19.2	May-December (2018)	−1.10–0.78 (PC-rich) −0.07–0.82 (PE-rich)	This study
Baltic Sea (Tvärminne Långskär station)	0.7–23.3	All seasons (1988)	−0.07–0.47	Kuosa, 1991
North West Atlantic (Martha’s Vineyard Observatory)	−2–22	All seasons (2003–2019)	−0.6–0.6	Hunter-Cevera et al., 2019
Mediterranean Sea (Bay of Blanes)	11–24	Feb-Jan (1996–1997)	0.2–1.5	Agawin et al., 1998
North Atlantic Ocean (Cape Hatteras, stations P1 and P2)	–	Jul-Aug (1984)	0.01–0.56	Campbell and Carpenter, 1986
North Atlantic Ocean (Skidaway Institute of Oceanography)	28.5–31.2	Jun-Aug (2017)	−0.88–1.02	Anderson et al., 2018
North Pacific Subtropical Gyre (Kaneohe Bay, Hawaii)	–	Sep (1982)	1.03–1.84	Landry et al., 1984
North West Indian Ocean (R.R.S. Charles Darwin, Gulf of Oman and South east of the Arabian peninsula)	–	Sep-Oct (1986)	−0.21–0.15	Burkill et al., 1993
South East Pacific (RV Southern Surveyor, Australia)	14.3–22.5	Oct (2010)	−0.45–0.32	Doblin et al., 2016
**PPE**				
Baltic Sea (K-station)	6.8–19.2	May-Dec (2018)	−0.01–0.91	This study
North Atlantic Ocean (Skidaway Institute of Oceanography)	28.5–31.2	Jun-Aug (2017)	−0.52–1.61	Anderson et al., 2018

PE-rich and PC-rich growth patterns and their response to nutrients could be divided into two periods: from May to July and from end-August to October/December. During the first period, PE-rich apparent net growth rates ranged from −0.01 to 0.82 day^–1^ while in the second period they were near zero (Table 1). During the first period, PE-rich growth was limited exclusively by nitrogen with a preference for NH_4_ over NO_3_ except on May 9th and June 19th (Supplementary Tables 3, 4). On May 9th, apparent net growth rates were co-limited by nitrogen (with a preference for NO_3_ over NH_4_) and phosphorus (Supplementary Tables 3, 4). On June 19th, the NO_3_ treatment showed significantly lower rates than the control. During the second period, the growth was limited by nitrogen at the end of September (preference for NH_4_) and at the beginning of October (preference for NO_3_). At < 15°C, NO_3_ was the preferred form of nitrogen, while at >15°C NH_4_ was preferred (Figure 1A and Table 1). PC-rich growth showed opposite dynamics to PE-rich. In the first period, PC-rich had poor apparent net growth rates while in the second period apparent net growth rates ranged from 0.34 to 0.78 day^–1^ (Table 1). During the first period, PC-rich growth was co-limited by nitrogen (with preference for NH_4_) and PO_4_ on June 4th and limited by nitrogen (with preference for NH_4_) on July 3rd (Supplementary Tables 3, 5). During the second period, growth was co-limited by nitrogen (both NO_3_ and NH_4_) and PO_4_ during October. At < 15°C, both NO_3_ and NH_4_ yielded a similar increase in apparent net growth rates compared to the control, while at >15°C, NH_4_ was the preferred form of nitrogen for PC-rich (Figure 1A and Table 1).

Photosynthetic picoeukaryotes apparent net growth rates were low in summer and high in spring and autumn (Table 1). During the two experiments in May, PPE apparent net growth rates ranged from 0.40 to 0.52 day^–1^ and increased significantly with PO_4_ addition and NH_4_ addition on the 6th and 20th of May, respectively (Supplementary Tables 3, 6). In the period from June to August, apparent net growth rates decreased from 0.33 to −0.01 day^–1^ and the addition of NO_3_, NH_4_, and PO_4_ reduced apparent net growth rates significantly compared to the control. During autumn, apparent net growth rates increased again to 0.56–0.91 day^–1^. PO_4_ limitation was observed during September. The highest apparent net growth rates for PPE were at temperatures 11–15°C. Nutrient addition at higher temperatures generally caused a significant decrease of the apparent net growth rates compared to the controls (Figure 1A and Table 1).

## Discussion

Picophytoplankton have a competitive advantage for nutrient uptake in oligotrophic environments where they contribute significantly to the total Chl *a* (Peña et al., 1990; Agawin et al., 2000; Durand et al., 2001). However, several observations have also pointed out the ecological relevance of picophytoplankton in coastal and eutrophic environments (Morán, 2007; Caroppo, 2015; Pulina et al., 2017; Paerl et al., 2020). Information about the seasonal abundance of picophytoplankton in the Baltic Sea is limited. As a consequence, picophytoplankton biomass contribution is frequently estimated using Chl *a* fractionation (Sondergaard et al., 1991; Stal et al., 1999, 2003; Ohlendieck et al., 2000; Tamm et al., 2018) or not included in the calculations (Uusitalo et al., 2013; Olofsson et al., 2020). At the K-station during 2018, picocyanobacteria were present throughout the year, with maximum abundances during summer (4.7 × 10^5^ cells mL^–1^). These numbers were comparable to other observations in the Baltic Sea Proper during summer (1.5 × 10^5^ to ∼5.5 × 10^5^ cells mL^–1^) (Albertano et al., 1997; Mazur-Marzec et al., 2013), suggesting that picocyanobacteria abundances at the coast are as high as in offshore locations. PE-rich cell abundance during spring at <10°C was notably higher than previous reports in the Baltic Sea and other temperate ecosystems (Kuosa, 1991; Hunter-Cevera et al., 2019). Similar abundances have also been recorded in the southern Baltic Proper (Mazur-Marzec et al., 2013) in line with observations of positive growth of *Synechococcus* at <2°C (Paulsen et al., 2016), suggesting that picocyanobacteria is adapted to low temperatures in the Baltic Sea. PPE abundance reached 1.1 × 10^5^ cells mL^–1^ during spring, around two orders of magnitude higher than the maximum abundances reported from the Gulf of Finland (Kuosa, 1991), and one order of magnitude higher than the maximum observed in other estuaries (Rajaneesh et al., 2015; Mohan et al., 2016). The current study (K-station) highlights the contribution of picocyanobacteria and PPE to the total phytoplankton biomass in the estuarine and eutrophic coastal Baltic Sea over an annual cycle.

Picophytoplankton is composed of multiple populations spanning diverse physiological adaptations and ranges (Rajaneesh et al., 2015; Tamm et al., 2018). Recent studies, in other estuarine and coastal areas, have separated *Synechococcus*/*Cyanobium* into pigment-based populations (PE-rich and PC-rich), suggesting differences in distribution patterns between the groups (Mitbavkar et al., 2015; Li et al., 2019; Paerl et al., 2020). In this study, the three populations, PE-rich, PC-rich, and PPE, had significant differences in regard to (1) seasonal dynamics, (2) biomass contribution, (3) temperature regimes, and (4) nutrient limitation. These results emphasize the importance of high-resolution studies of ecological relevant populations in order to understand the dynamics of the genetic and physiologically diverse picophytoplankton (Not et al., 2009; Bertos-Fortis et al., 2016).

Seasonal variations in the PE-rich and PC-rich contributions to the picocyanobacteria community have previously been observed in tropical and subtropical estuaries (Liu et al., 2014; Rajaneesh et al., 2015; Jiang et al., 2016; Li et al., 2019). At the K-station, PE-rich and PC-rich abundances increased during spring and peaked during early summer. During this period, PE-rich dominated the picocyanobacteria community (up to 99%). This was consistent with previous observations of PE-rich dominance in the Baltic Sea during the spring–summer period (Mazur-Marzec et al., 2013; Larsson et al., 2014; Tamm et al., 2018). During the autumn, PE-rich abundance declined, resulting in PC-rich dominance. The reduction in irradiance from summer to autumn can cause a transition from PE-rich to PC-rich (Stomp et al., 2007). However, other environmental factors such as nutrient concentration may also influence the picocyanobacterial community composition (Mitbavkar et al., 2015). PPE increase in abundance is thought to be related to low temperatures and high nutrient concentration (Kuosa, 1991; Otero-Ferrer et al., 2018). Our observations showed that PPE abundances peaked during spring, at 11–15°C. The low PPE abundances during autumn contrast with the peak abundances observed by Kuosa (1991) during the same period. These patterns could be explained by the long-lasting summer of 2018 (Humborg et al., 2019), which could have shifted PPE favorable temperatures to later in the autumn while concurrent light limitation may have restricted PPE growth (Fowler et al., 2020).

The biomass estimates at the K-station confirmed that the pico-fraction (<2 μm) is a major contributor to the total phytoplankton biomass, in line with previous recordings in the Baltic Sea based on Chl *a* measurements (Sondergaard et al., 1991; Stal et al., 1999, 2003; Ohlendieck et al., 2000; Tamm et al., 2018), and can dominate the phytoplankton community during spring, early summer, and autumn. Resolving the biomass estimates into the three populations reveal that on an annual basis, PPE was the main contributor to the phytoplankton community except during the bloom of N_2_-fixers at the end of July (>18°C). This highlights the importance of PPE in coastal environments (Worden et al., 2004). A high contribution of both PE-rich and PC-rich co-occur with high temperatures, in line with previous studies (Stal et al., 2003). A community composition shift from PPE to picocyanobacteria can have large impacts on the microbial food web and should be systematically included in future phytoplankton biomass studies and carbon flux models (Schmidt et al., 2020).

Range adaptation to temperature showed different patterns among the picophytoplankton groups. The highest apparent net growth rates were observed during spring and beginning of summer at 10°C and 17–19°C for PE-rich and at 11–15°C for PPE. The temperature range for PC-rich was 13–16°C, as the highest apparent net growth rates were during autumn. The apparent net growth rates of picocyanobacteria were in line with previous observations in temperate ecosystems (Table 2). Similarly to the high-resolution growth dynamic study by Hunter-Cevera et al. (2016), in this Baltic Sea study, apparent net growth showed no increase at >16–17°C. However, higher apparent net growth rates of *Synechococcus* (up to 1.84 day^–1^) have been reported in warmer climate (Table 2). Thus, an increase in temperature due to climate change might result in an increase of *Synechococcus* growth at higher latitudes (Stawiarski et al., 2016). The apparent growth calculations did not account for grazing or viral lysis. Grazing by ciliates and flagellates (2–20 μm) is considered an important top-down control on picophytoplankton (Grinienë et al., 2016). However, some observations have reported that grazing fails to control picocyanobacterial blooms, particularly in nutrient-rich environments (Kuuppo et al., 2003; Samuelsson, 2003). On the other hand, viral lysis can also influence picophytoplankton growth (Tsai et al., 2015) but may be a minor factor, particularly for PPE (Tsai et al., 2018).

The bioassays showed that NH_4_ was the preferred form of nitrogen for picophytoplankton. The preference of NH_4_ over NO_3_ for *Synechococcus/Cyanobium* has been extensively documented (Moore et al., 2002; Berthelot et al., 2018; Klawonn et al., 2019; Tan et al., 2019). This study shows that both PE-rich and PC-rich can use NO_3_ and NH_4_ at low temperature but showed preference for NH_4_ at higher temperatures (>15°C–17°C). This is in line with the effect of temperature on nitrogen assimilation enzymatic pathways (Glibert et al., 2016). The assimilation of NH_4_ generally occurs through the GS-GOGAT pathway, while NO_3_ uptake depends on the enzyme nitrate reductase (NR). GS-GOGAT is positively correlated with temperature, while NR is negatively correlated (Glibert et al., 2016). As a result, NH_4_ assimilation will be higher than NO_3_ assimilation at high temperatures. Thus, the uptake of NH_4_ is an advantageous adaptation for *Synechococcus/Cyanobium* to compete with other NO_3_ specialists such as diatoms under nitrogen limitation during the warm periods (Glibert et al., 2016). It should be noted that our results shows that Baltic Sea PE-rich were better adapted to high temperatures than PC-rich, and as a consequence PE-rich may benefit more from NH_4_ uptake. In coastal areas and shallow water ecosystems (<50 m depth), NH_4_ from benthic or riverine origin can be the main nitrogen source for the phytoplankton community (Herbert, 1999; Klawonn et al., 2019), which could benefit PE-rich at high temperatures. In line with the observations by Berthelot et al. (2018), PPE growth increased in NH_4_ addition treatments during nitrogen limitation. However, nutrient additions outside of the temperature range resulted in significant reductions of the apparent net growth rates of PPE. This was likely because nutrient addition in a nutrient-limited system can favor competitors better adapted for high temperatures than PPE such as PE-rich.

This study provides an annual high-resolution description of picophytoplankton abundance and dynamics in the coastal Baltic Sea Proper. It also investigates apparent net growth rates and nutrient limitation of three functional picophytoplankton groups. In this study, PE-rich, PC-rich, and PPE showed different seasonal dynamics defined by different temperature ranges and nutrient limitation. To understand the dynamics of genotypes, future research should include the molecular diversity within each group. This study shows, for the first time, the contribution and importance of picophytoplankton over a full annual cycle and situates PPE as one of the most important components of the phytoplankton community in terms of biomass especially during spring and early summer. The results further suggest that in eutrophic coastal systems where NH_4_ is the main nitrogen species (agricultural landscape), PE-rich will be favored over PPE. This effect could be further magnified during earlier and more extensive blooms of N_2_-fixing cyanobacteria that are projected as a consequence of global warming (Neumann et al., 2012; Andersson et al., 2015). Such events could favor PE-rich over PC-rich and PPE, leading to picophytoplankton community shifts having consequences on the contribution to the total carbon biomass in coastal areas.

## Data Availability Statement

The original contributions presented in the study are included in the article/Supplementary Material, further inquiries can be directed to the corresponding author/s.

## Author Contributions

CL, HF, and JA conceived the study. JA and HF conducted the field sampling and bioassay experiments. JA performed the laboratory and data analysis and plotted the figures. JA, CL, and HF wrote and edited the manuscript. All authors contributed to the article and approved the submitted version.

## Conflict of Interest

The authors declare that the research was conducted in the absence of any commercial or financial relationships that could be construed as a potential conflict of interest.

## Publisher’s Note

All claims expressed in this article are solely those of the authors and do not necessarily represent those of their affiliated organizations, or those of the publisher, the editors and the reviewers. Any product that may be evaluated in this article, or claim that may be made by its manufacturer, is not guaranteed or endorsed by the publisher.

## References

[B1] Agawin N. S. R. Duarte C. M. Agustí S. (1998). Growth and abundance of *Synechococcus* sp. in a Mediterranean Bay: seasonality and relationship with temperature. *Mar. Ecol. Prog. Ser.* 170 45–53. 10.3354/meps170045

[B2] Agawin N. S. R. Duarte C. M. Agustí S. (2000). Nutrient and temperature control of the contribution of picoplankton to phytoplankton biomass and production. *Limnol. Oceanogr.* 45 591–600. 10.4319/lo.2000.45.3.0591

[B3] Albertano P. Di Somma D. Capucci E. (1997). Cyanobacterial picoplankton from the central Baltic Sea: cell size classification by image analyzed fluorescence microscopy. *J. Plankton Res.* 19 1405–1416. 10.1093/plankt/19.10.1405

[B4] Andersson A. Meier H. E. M. Ripszam M. Rowe O. Wikner J. Haglund P. (2015). Projected future climate change and Baltic Sea ecosystem management. *AMBIO* 44 345–356. 10.1007/s13280-015-0654-8 26022318 PMC4447695

[B5] Anderson S. R. Diou-Cass Q. P. Harvey E. L. (2018). Short-term estimates of phytoplankton growth and mortality in a tidal estuary. *Limnol. Oceanogr.* 63 2411–2422. 10.1002/lno.10948

[B6] Berthelot H. Duhamel S. L’Helguen S. Maguer J. F. Wang S. Cetinić I. (2018). NanoSIMS single cell analyses reveal the contrasting nitrogen sources for small phytoplankton. *ISME J.* 13 651–662. 10.1038/s41396-018-0285-8 30323264 PMC6461931

[B7] Bertos-Fortis M. Farnelid H. M. Lindh M. V. Casini M. Andersson A. Pinhassi J. (2016). Unscrambling cyanobacteria community dynamics related to environmental factors. *Front. Microbiol.* 7:625. 10.3389/fmicb.2016.00625 27242679 PMC4860504

[B8] Burkill P. H. Leakey R. J. G. Owens N. J. P. Mantoura R. F. C. (1993). Synechococcus and its importance to the microbial foodweb of the northwestern Indian Ocean. *Deep Sea Res. Part II Top. Stud. Oceanogr.* 40 773–782. 10.1016/0967-0645(93)90057-T

[B9] Callieri C. Stockner J. G. (2002). Freshwater autotrophic picoplankton: a review. *J. Limnol.* 61 1–14. 10.4081/jlimnol.2002.1

[B10] Calvo-Díaz A. Morán X. A. G. Nogueira E. Bode A. Varela M. (2004). Picoplankton community structure along the northern Iberian continental margin in late winter-early spring. *J. Plankton Res.* 26 1069–1081. 10.1093/plankt/fbh098

[B11] Campbell L. Carpenter E. (1986). Estimating the grazing pressure of heterotrophic nanoplankton on *Synechococcus* spp. using the sea water dilution and selective inhibitor techniques. *Mar. Ecol. Prog. Ser.* 33 121–129. 10.3354/meps033121

[B12] Campbell L. Carpenter E. J. (1987). Characterization of phycoerythrin-containing *Synechococcus* spp. populations by immunofluorescence. *J. Plankton Res.* 9 1167–1181. 10.1093/plankt/9.6.1167

[B13] Caroppo C. (2015). Ecology and biodiversity of picoplanktonic cyanobacteria in coastal and brackish environments. *Biodivers. Conserv.* 24 949–971. 10.1007/s10531-015-0891-y

[B14] Celepli N. Sundh J. Ekman M. Dupont C. L. Yooseph S. Bergman B. (2017). Meta-omic analyses of Baltic Sea cyanobacteria: diversity, community structure and salt acclimation. *Environ. Microbiol.* 19 673–686. 10.1111/1462-2920.13592 27871145

[B15] Doblin M. A. Petrou K. Sinutok S. Seymour J. R. Messer L. F. Brown M. V. (2016). Nutrient uplift in a cyclonic eddy increases diversity, primary productivity and iron demand of microbial communities relative to a western boundary current. *PeerJ.* 4:e1973. 10.7717/peerj.1973 27168982 PMC4860325

[B16] Durand M. D. Olson R. J. Chisholm S. W. (2001). Phytoplankton population dynamics at the Bermuda Atlantic Time-series station in the Sargasso Sea. *Deep Sea Res. II Top. Stud. Oceanogr.* 48 1983–2003. 10.1016/S0967-0645(00)00166-1

[B17] Edler L. (1979). *Recommendations on Methods for Marine Biological Studies in the Baltic Sea: Phytoplankton and Chlorophyll.* Stockholm: University of Stockholm.

[B18] Flombaum P. Gallegos J. L. Gordillo R. A. Rincón J. Zabala L. L. Jiao N. (2013). Present and future global distributions of the marine Cyanobacteria *Prochlorococcus* and *Synechococcus*. *Proc. Natl. Acad. Sci. U.S.A.* 110 9824–9829. 10.1073/pnas.1307701110 23703908 PMC3683724

[B19] Fowler B. L. Neubert M. G. Hunter-Cevera K. R. Olson R. J. Shalapyonok A. Solow A. R. (2020). Dynamics and functional diversity of the smallest phytoplankton on the Northeast US Shelf. *Proc. Natl. Acad. Sci. U.S.A.* 117:12221. 10.1073/pnas.1918439117 32414929 PMC7275697

[B20] Glibert P. M. Wilkerson F. P. Dugdale R. C. Raven J. A. Dupont C. L. Leavitt P. R. (2016). Pluses and minuses of ammonium and nitrate uptake and assimilation by phytoplankton and implications for productivity and community composition, with emphasis on nitrogen-enriched conditions. *Limnol. Oceanogr.* 61 165–197. 10.1002/lno.10203

[B21] Grinienë E. Šulčius S. Kuosa H. (2016). Size-selective microzooplankton grazing on the phytoplankton in the Curonian Lagoon (SE Baltic Sea). *Oceanologia* 58 292–301. 10.1016/j.oceano.2016.05.002

[B22] Hajdu S. Höglander H. Larsson U. (2007). Phytoplankton vertical distributions and composition in Baltic Sea cyanobacterial blooms. *Harmful Algae* 6 189–205. 10.1016/j.hal.2006.07.006

[B23] Hammill D. (2019). *CytoRSuite. R package, version 0.9.9.*

[B24] Haverkamp T. Acinas S. G. Doeleman M. Stomp M. Huisman J. Stal L. J. (2008). Diversity and phylogeny of Baltic Sea picocyanobacteria inferred from their ITS and phycobiliprotein operons. *Environ. Microbiol.* 10 174–188. 10.1111/j.1462-2920.2007.01442.x 17903216

[B25] HELCOM Phytoplankton Expert Group (2013). *Phytoplankton Biovolume and Carbon Content.* Available online at: https://www.ices.dk/data/Documents/ENV/PEG_BVOL.zip (accessed August 6, 2019).

[B26] Herbert R. A. (1999). Nitrogen cycling in coastal marine ecosystems. *FEMS Microbiol. Rev.* 23 563–590. 10.1016/S0168-6445(99)00022-4 10525167

[B27] Herlemann D. P. Labrenz M. Jürgens K. Bertilsson S. Waniek J. J. Andersson A. F. (2011). Transitions in bacterial communities along the 2000 km salinity gradient of the Baltic Sea. *ISME J.* 5 1571–1579. 21472016 10.1038/ismej.2011.41 PMC3176514

[B28] Hu Y. O. O. Karlson B. Charvet S. Andersson A. F. (2016). Diversity of pico- to mesoplankton along the 2000 km salinity gradient of the baltic sea. *Front. Microbiol.* 7:679. 10.3389/fmicb.2016.00679 27242706 PMC4864665

[B29] Humborg C. Geibel M. C. Sun X. McCrackin M. Mörth C. M. Stranne C. (2019). High emissions of carbon dioxide and methane from the coastal Baltic Sea at the end of a summer heat wave. *Front. Mar. Sci.* 6:493. 10.3389/fmars.2019.00493

[B30] Hunter-Cevera K. R. Neubert M. G. Olson R. J. Shalapyonok A. Solow A. R. Sosik H. M. (2019). Seasons of Syn. *Limnol. Oceanogr.* 65 1–18. 10.1002/lno.11374 PMC7319482 32612307

[B31] Hunter-Cevera K. R. Post A. F. Peacock E. E. Sosik H. M. (2016). Diversity of *Synechococcus* at the Martha’s vineyard coastal observatory: insights from culture isolations, clone libraries, and flow cytometry. *Microb. Ecol.* 71 276–289. 10.1007/s00248-015-0644-1 26233669 PMC4728178

[B32] Jespersen A.-M. Christoffersen K. (1987). Measurements of chlorophyll a from phytoplankton using ethanol as extraction solvent. *Arch. Hydrobiol.* 109:445–454.

[B33] Jiang T. Chai C. Wang J. Zhang L. Cen J. Lu S. (2016). Temporal and spatial variations of abundance of phycocyanin- and phycoerythrin-rich Synechococcus in Pearl River Estuary and adjacent coastal area. *J. Ocean Univ. China* 15 897–904. 10.1007/s11802-016-3011-z

[B34] Klawonn I. Bonaglia S. Whitehouse M. J. Littmann S. Tienken D. Kuypers M. M. M. M. (2019). Untangling hidden nutrient dynamics: rapid ammonium cycling and single-cell ammonium assimilation in marine plankton communities. *ISME J.* 13 1960–1974. 10.1038/s41396-019-0386-z 30911131 PMC6776039

[B35] Komárek J. Kopeckı J. Cepák V. (1999). Generic characters of the simplest cyanoprokaryotes Cyanobium, *Cyanobacterium* and *Synechococcus*. *Cryptogam. Algol.* 20 209–222. 10.1016/S0181-1568(99)80015-4

[B36] Kuosa H. (1991). Picoplanktonic algae in the northern Baltic Sea: seasonal dynamics and flagellate grazing. *Mar. Ecol. Prog. Ser.* 73 269–276. 10.3354/meps073269

[B37] Kuuppo P. Samuelsson K. Lignell R. Seppälä J. Tamminen T. Andersson A. (2003). Fate of increased production in late-summer plankton communities due to nutrient enrichment of the Baltic Proper. *Aquat. Microb. Ecol.* 32 47–60. 10.3354/ame032047

[B38] Landry M. Haas L. Fagerness V. (1984). Dynamics of microbial plankton communities: experiments in Kaneohe Bay, Hawaii. *Mar. Ecol. Prog. Ser.* 16 127–133. 10.3354/meps016127

[B39] Lagus A. Suomela J. Helminen H. Sipura J. (2007). Impacts of nutrient enrichment and sediment on phytoplankton community structure in the northern Baltic Sea. *Hydrobiologia* 579 351–368. 10.1007/s10750-006-0491-7

[B40] Larsson J. Celepli N. Ininbergs K. Dupont C. L. Yooseph S. Bergman B. (2014). Picocyanobacteria containing a novel pigment gene cluster dominate the brackish water Baltic Sea. *ISME J.* 8 1892–1903. 10.1038/ismej.2014.35 24621524 PMC4139726

[B41] Legrand C. Fridolfsson E. Bertos-Fortis M. Lindehoff E. Larsson P. Pinhassi J. (2015). Interannual variability of phyto-bacterioplankton biomass and production in coastal and offshore waters of the Baltic Sea. *AMBIO* 44 427–438. 10.1007/s13280-015-0662-8 26022325 PMC4447688

[B42] Li J. Chen Z. Jing Z. Zhou L. Li G. Ke Z. (2019). Synechococcus bloom in the Pearl River Estuary and adjacent coastal area-With special focus on flooding during wet seasons. *Sci. Total Environ.* 692 769–783. 10.1016/j.scitotenv.2019.07.088 31539984

[B43] Liu H. Jing H. Wong T. H. C. Chen B. (2014). Co-occurrence of phycocyanin- and phycoerythrin-rich Synechococcus in subtropical estuarine and coastal waters of Hong Kong. *Environ. Microbiol. Rep.* 6 90–99. 10.1111/1758-2229.12111 24596266

[B44] Magazzu G. Decembrini F. (1995). Primary production, biomass and abundance of phototrophic picoplankton in the Mediterranean Sea: a review. *Aquat. Microb. Ecol.* 9 97–104. 10.3354/ame009097

[B45] Mazur-Marzec H. Sutryk K. Kobos J. Hebel A. Hohlfeld N. Błaszczyk A. (2013). Occurrence of cyanobacteria and cyanotoxin in the Southern Baltic Proper. *Filamentous cyanobacteria* versus single-celled picocyanobacteria. *Hydrobiologia* 701 235–252. 10.1007/s10750-012-1278-7

[B46] Mitbavkar S. Patil J. S. Rajaneesh K. M. (2015). Picophytoplankton as tracers of environmental forcing in a tropical monsoonal bay. *Microb. Ecol.* 70 659–676. 10.1007/s00248-015-0599-2 25851443

[B47] Mohan A. P. Jyothibabu R. Jagadeesan L. Lallu K. R. Karnan C. (2016). Summer monsoon onset-induced changes of autotrophic pico- and nanoplankton in the largest monsoonal estuary along the west coast of India. *Environ. Monit. Assess.* 188:93. 10.1007/s10661-016-5096-7 26780412

[B48] Moore L. R. Post A. F. Rocap G. Chisholm S. W. (2002). Utilization of different nitrogen sources by the marine cyanobacteria *Prochlorococcus* and *Synechococcus*. *Limnol. Oceanogr.* 47 989–996. 10.4319/lo.2002.47.4.0989

[B49] Morán X. A. G. (2007). Annual cycle of picophytoplankton photosynthesis and growth rates in a temperate coastal ecosystem: a major contribution to carbon fluxes. *Aquat. Microb. Ecol.* 49 267–279. 10.3354/ame01151

[B50] Neumann T. Eilola K. Gustafsson B. Müller-Karulis B. Kuznetsov I. Meier H. E. M. (2012). Extremes of temperature, oxygen and blooms in the baltic sea in a changing climate. *AMBIO* 41 574–585. 10.1007/s13280-012-0321-2 22926880 PMC3428485

[B51] Not F. del Campo J. Balagué V. de Vargas C. Massana R. (2009). New insights into the diversity of marine picoeukaryotes. *PLoS One* 4:e7143. 10.1371/journal.pone.0007143 19787059 PMC2747013

[B52] Ohlendieck U. Stuhr A. Siegmund H. (2000). Nitrogen fixation by diazotrophic cyanobacteria in the Baltic Sea and transfer of the newly fixed nitrogen to picoplankton organisms. *J. Mar. Syst.* 25 213–219. 10.1016/S0924-7963(00)00016-6

[B53] Olenina I. Hajdu S. Edler L. Andersson A. Wasmund N. Busch S. (2006). Biovolumes and size-classes of phytoplankton in the Baltic Sea. *Connaissance Des. Arts* 607 110–114.

[B54] Olofsson M. Hagan J. G. Karlson B. Gamfeldt L. (2020). Large seasonal and spatial variation in nano- and microphytoplankton diversity along a Baltic Sea—North Sea salinity gradient. *Sci. Rep.* 10:17666. 10.1038/s41598-020-74428-8 33077730 PMC7572517

[B55] Otero-Ferrer J. L. Cermeño P. Bode A. Fernández-Castro B. Gasol J. M. Morán X. A. G. (2018). Factors controlling the community structure of picoplankton in contrasting marine environments. *Biogeosciences* 15 6199–6220. 10.5194/bg-15-6199-2018

[B56] Paerl R. W. Venezia R. E. Sanchez J. J. Paerl H. W. (2020). Picophytoplankton dynamics in a large temperate estuary and impacts of extreme storm events. *Sci. Rep.* 10:22026. 10.1038/s41598-020-79157-6 33328574 PMC7744581

[B57] Partensky F. Hess W. R. Vaulot D. (1999). Prochlorococcus, a marine photosynthetic prokaryote of global significance. *Microbiol. Mol. Biol. Rev.* 63 106–127. 10.1128/MMBR.63.1.106-127.1999 10066832 PMC98958

[B58] Paulsen M. L. Doré H. Garczarek L. Seuthe L. Müller O. Sandaa R. A. (2016). *Synechococcus* in the Atlantic Gateway to the Arctic Ocean. *Front. Mar. Sci.* 3:191. 10.3389/fmars.2016.00191

[B59] Peña M. A. Lewis M. R. Harrison W. G. (1990). Primary productivity and size structure of phytoplankton biomass on a transect of the equator at 135°W in the Pacific Ocean. *Deep Sea Res.* 19 295–315. 10.1016/0198-0149(90)90129-J

[B60] Pittera J. Humily F. Thorel M. Grulois D. Garczarek L. Six C. (2014). Connecting thermal physiology and latitudinal niche partitioning in marine *Synechococcus*. *ISME J.* 8 1221–1236. 10.1038/ismej.2013.228 24401861 PMC4030225

[B61] Ploug H. Adam B. Musat N. Kalvelage T. Lavik G. Wolf-Gladrow D. (2011). Carbon, nitrogen and O2 fluxes associated with the cyanobacterium *Nodularia spumigena* in the Baltic Sea. *ISME J.* 5 1549–1558. 10.1038/ismej.2011.20 21390075 PMC3160678

[B62] Pulina S. Satta C. T. Padedda B. M. Bazzoni A. M. Sechi N. Lugliè A. (2017). Picophytoplankton seasonal dynamics and interactions with environmental variables in three mediterranean coastal lagoons. *Estuar. Coasts* 40 469–478. 10.1007/s12237-016-0154-5

[B63] R Core Team (2019). *R: A Language and Environment for Statistical Computing. R version 3.5.1.* Vienna: R Foundation for Statistical Computing.

[B64] Rajaneesh K. M. Mitbavkar S. Anil A. C. Sawant S. S. (2015). *Synechococcus* as an indicator of trophic status in the Cochin backwaters, west coast of India. *Ecol. Indic.* 55 118–130. 10.1016/j.ecolind.2015.02.033

[B65] Rii Y. M. Karl D. M. Church M. J. (2016). Temporal and vertical variability in picophytoplankton primary productivity in the North Pacific Subtropical Gyre. *Mar. Ecol. Prog. Ser.* 562 1–18. 10.3354/meps11954

[B66] Samuelsson K. (2003). *Mechanisms Structuring the Pelagic Microbial Food Web - Importance of Resources and Predation.* Doctoral thesis. Västerbottens län: Department of Ecology and Environmental Science Umeå University.

[B67] Sánchez-Baracaldo P. Hayes P. K. Blank C. E. (2005). Morphological and habitat evolution in the Cyanobacteria using a compartmentalization approach. *Geobiology* 3 145–165. 10.1111/j.1472-4669.2005.00050.x

[B68] Sathicq M. B. Unrein F. Gómez N. (2020). Recurrent pattern of picophytoplankton dynamics in estuaries around the world: the case of Río de la Plata. *Mar. Environ. Res.* 161:105136. 10.1016/j.marenvres.2020.105136 32971494

[B69] Schmidt K. Birchill A. J. Atkinson A. Brewin R. J. W. Clark J. R. Hickman A. E. (2020). Increasing picocyanobacteria success in shelf waters contributes to long-term food web degradation. *Glob. Change Biol.* 26 5574–5587. 10.1111/gcb.15161 32506810

[B70] Sondergaard M. Jensen L. M. Aertebjerg G. (1991). Picoalgae in Danish coastal waters during summer stratification. *Mar. Ecol. Prog. Ser.* 79 139–149. 10.3354/meps079139

[B71] Stal L. Albertano P. Bergman B. Von Bröckel K. Gallon J. R. Hayes P. K. (2003). BASIC: Baltic Sea cyanobacteria. An investigation of the structure and dynamics of water blooms of cyanobacteria in the Baltic Sea - Responses to a changing environment. *Cont. Shelf Res.* 23 1695–1714. 10.1016/j.csr.2003.06.001

[B72] Stal L. Staal M. Villbrandt M. (1999). Nutrient control of cyanobacterial blooms in the Baltic Sea. *Aquat. Microb. Ecol.* 18 165–173. 10.3354/ame018165

[B73] Stal L. Walsby A. (2000). Photosynthesis and nitrogen fixation in a cyanobacterial bloom in the baltic sea. *Eur. J. Phycol.* 35 97–108. 10.1080/09670260010001735681

[B74] Stawiarski B. Buitenhuis E. T. Quéré C. L. (2016). The physiological response of picophytoplankton to temperature and its model representation. *Front. Mar. Sci.* 3:164. 10.3389/fmars.2016.00164

[B75] Stomp M. Huisman J. Vörös L. Pick F. R. Laamanen M. Haverkamp T. (2007). Colourful coexistence of red and green picocyanobacteria in lakes and seas. *Ecol. Lett.* 10 290–298. 10.1111/j.1461-0248.2007.01026.x 17355568

[B76] Tamm M. Laas P. Freiberg R. Nõges P. Nõges T. (2018). Parallel assessment of marine autotrophic picoplankton using flow cytometry and chemotaxonomy. *Sci. Total Environ.* 625 185–193. 10.1016/j.scitotenv.2017.12.234 29289004

[B77] Tan X. Gu H. Ruan Y. Zhong J. Parajuli K. Hu J. (2019). Effects of nitrogen on interspecific competition between two cell-size cyanobacteria: *Microcystis aeruginosa* and *Synechococcus* sp. *Harmful Algae* 89:101661. 10.1016/j.hal.2019.101661 31672227

[B78] Tsai A. Y. Gong G. C. Chung C. C. Huang Y. T. (2018). Different impact of nanoflagellate grazing and viral lysis on *Synechococcus* spp. and picoeukaryotic mortality in coastal waters. *Estuar. Coast.* 209 1–6. 10.1016/j.ecss.2018.05.012

[B79] Tsai A. Y. Gong G. C. Huang Y. W. Chao C. F. (2015). Estimates of bacterioplankton and *Synechococcus* spp. mortality from nanoflagellate grazing and viral lysis in the subtropical Danshui River estuary. *Estuar. Coast.* 153 54–61. 10.1016/j.ecss.2014.11.032

[B80] Uitz J. Claustre H. Gentili B. Stramski D. (2010). Phytoplankton class-specific primary production in the world’s oceans: seasonal and interannual variability from satellite observations. *Glob. Biogeochem. Cycles* 24:GB3016. 10.1029/2009GB003680

[B81] Utermöhl H. (1958). Zur vervollkommnung der quantitativen phytoplankton- methodik. *Mitteilung Int. Vereinigung Fuer Theor. Amgewandte Limnol.* 9 1–38.

[B82] Uusitalo L. Fleming-lehtinen V. Ha H. Jaanus A. Ha S. (2013). A novel approach for estimating phytoplankton biodiversity. *ICES J. Mar. Sci.* 70 408–417.

[B83] Valderrama J. C. (1995). “Methods of nutrient analysis,” in *Manual on Harmful Marine Microalgae*, eds Hallagraeff G. M. Anderson D. M. Cembella A. D. (Mumbai: IOC Manuals and Guides), 251–268.

[B84] Vörös L. Callieri C. V-Balogh K. Bertoni R. (1998). Freshwater picocyanobacteria along a trophic gradient and light quality range. *Hydrobiologia* 369-370 117–125. 10.1007/978-94-017-2668-9_10

[B85] Worden A. Z. Nolan J. K. Palenik B. (2004). Assessing the dynamics and ecology of marine picophytoplankton: the importance of the eukaryotic component. *Limnol. Oceanogr.* 49 168–179. 10.4319/lo.2004.49.1.0168

