# Supplementary material for: Seasonality of Coastal Picophytoplankton Growth, Nutrient Limitation, and Biomass Contribution
Source: Front Microbiol. 2021 Dec 6;12:786590. doi: 10.3389/fmicb.2021.786590 (PMC8685431; doi:10.3389/fmicb.2021.786590)
Supplement: Supplementary file 1 [file Data_Sheet_1.doc]

Supplementary Material

# Seasonality of coastal picophytoplankton growth, nutrient limitation and biomass contribution

Javier Alegria Zufia^1^, Hanna Farnelid^1^, Catherine Legrand^1,2*^

^1^Marine phytoplankton ecology and applications Laboratory(MPEA), Linnaeus University, Department of Biology and Environmental Science, Centre for Ecology and Evolution in Microbial Model Systems (EEMiS), Kalmar, Sweden

^2^School of Business, Innovation and Sustainability, Halmstad University, Halmstad, Sweden

# Supplementary information

## Flow cytometry identification of picophytoplankton groups

The three picophytoplankton functional groups phycoerythrin rich (PE-rich) *Synechococcus*, phycocyanin rich (PC-rich) picocyanobacteria and picoeukaryotes (PPE) were identified and counted using a CyFlow® Cube8 flow cytometer (Partec®, Germany; **Supplementary Figure S2**). For the cell characterization, four optical parameters were used at a logarithmic scale: Forward scatter (FSC) as a proxy for cell diameter, FL2 (590/50 nm, blue laser dependent) as a proxy for PE content, FL3 (675/50 nm, blue laser dependent) as a proxy for chlorophyll a (Chl a) and FL4 (675/50 nm, red laser dependent) as a proxy for PC content (Liu et al., 2014). The identification was performed as following:

PE-rich *Synechococcus* was gated on the FL2 vs FL3 scatterplot. PE-rich *Synechococcus* showed high FL2 signal and in the FL3 vs FSC scatterplot the events were close to the 1 µm diameter beads. PC-rich picocyanobacteria was gated in two steps: First, all the events with low FL2 signal on the FL2 vs FL4 scatterplot were gated to exclude events with high PE content. Then on the FL4 vs FSC scatterplot, events with high FL4 signal and a diameter close to the 1 µm beads was gated. PPE was gated on the FSC vs FL3 scatterplot. PPE showed high Chl a content (high FL3) and a diameter between the 1 and the 3 µm diameter beads. To avoid overlap with PE-rich, it was verified that the events in the PPE gate did not have a high PE (FL2) signal.

# Supplementary Figures

**Supplementary Figure S1.** Map of south Sweden and the Baltic Sea with the location of the K-station marked with a red ×.

**Supplementary Figure 2.** Flow cytometry cytograms illustrating the identification of the picophytoplankton groups PE-rich *Synechococcus* (red), PC-rich picocyanobacteria (blue), PPE (green) and 3 µm beads (purple) for (A) FCS vs FL3, (B) FL2 vs FL3, (C) FSC vs FL4 and (D) FL2 vs FL4.





**Supplementary Figure S3.** Bioassays experimental design The bioassay treatments (represented by the flasks) were set in triplicates and consisted on the addition of NH_4_ (200 µM), NO_3_ (200 µM), PO_4_ (10 µM), NO_3_ (200 µM)+PO_4_ (10 µM), NH_4_ (200 µM)+PO_4_ (10 µM) and controls without nutrient addition. Sampling from the treatments (represented by the black dots) was performed after the set up of the experiment (0h) and after 48h.


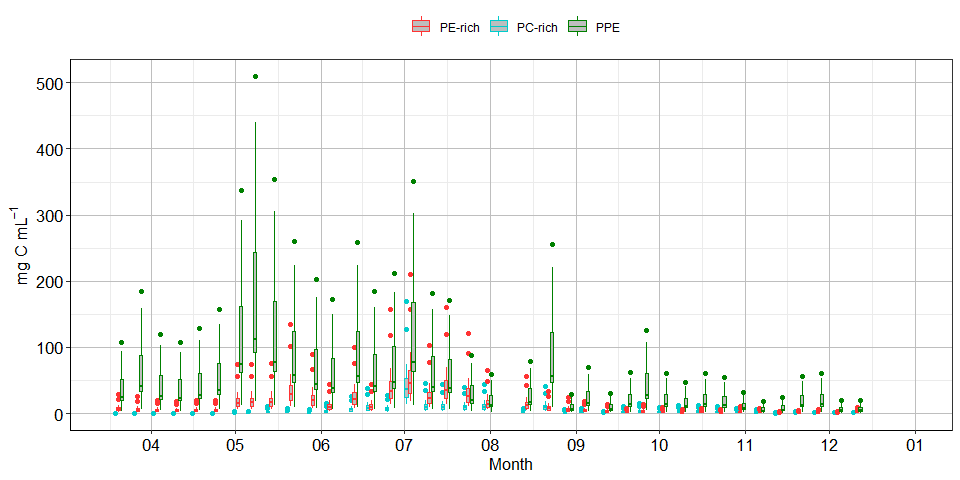


**Supplementary Figure S4.** K-station weekly carbon biomass for PE-rich *Synechococcus* (red), PC-rich picocyanobacteria (blue), PPE (green). The relative contribution of picophytoplankton based on the carbon biomass concentration was estimated for each value included in **Supplementary Table S1***.*

Supplementary Tables

Supplementary Table 1. Cell abundance to carbon biomass conversion factors (fg C cell^−1^) used to calculate the carbon biomass contribution of picophytoplankton (PE-rich, PC-rich and PPE) in the field observations in Fig. 3. The carbon biomass concentration of picophytoplankton was estimated by calculating the min-max range of relative contribution of PE-rich, PC-rich and PPE to total phytoplankton carbon biomass based on a combination of conversion factors estimated for carbon content collected from the literature.

| **Picocyanobacteria** | **Picoeukaryote** | **Reference** |
| --- | --- | --- |
| 250 fg C cell^−1^ |  | Kana et al., 1987 |
| 600 fg C cell^−1^ | 3800 ± 100 fg C cell^−1^ | Verity et al., 1992 |
|  | 800, 1360 fg C cell^−1^ | Montagnes et al., 1994 |
| 350 fg C cell^−1^ |  | Liu et al., 1999 |
|  | 4400 fg C cell^−1^ | Llewellyn et al., 2000 |
| 170 ±65 fg C cell^−1^ |  | Bertilsson et al., 2003 |
| 249 ± 21 fg C cell^−1^ |  | Fu et al., 2007 |
| 175 fg C cell^−1^ |  | Veldhuis et al., 1997 |
| 246 fg C cell^−1^ | 2108 fg C cell^−1^ | Campbell et al., 1994 |
| 112 fg C cell^−1^ |  | Durand et al., 2001 |
| 82 ± 8 fg C cell^−1^ | 530 ± 185 fg C cell^−1^ | Worden et al., 2004 |
| 105 fg C cell^−1^ |  | Sondergaard et al., 1991 |
| 800 fg C cell^−1^ |  | Edler, 1979 |
| 129 fg C cell^−1^ | 160 fg C cell^−1^ | Wei et al., 2019 |
| 120 fg C cell^−1^ | 829 fg C cell^−1^ | Paczkowska et al., 2020 |
| 294 fg C cell^−1^ |  | Cuhel et al., 1984 |
| 250 fg C cell^−1^ | 964 fg C cell^−1^ | Wei et al., 2020 |
| 157 fg C cell^−1^, 67 fg C cell^−1^ |  | Liu et al., 2007 |
| 103 fg C cell^−1^ |  | Zubkov et al., 1998 |

Supplementary Table 2. Bioassay in situ concentrations of NO_3_ and PO_4_ (µM) on the dates of the bioassay experiments as shown in Figure 1. The temperature presented is the average temperature during the incubation period. In the K-station NH_4_ typically ranges 0.02-2.52 µM (analysis performed according to Valderrama (1995)).

| **Bioassay date** | **NO_3_ (µM)** | **PO_4_ (µM)** | **Temperature (°C)** |
| --- | --- | --- | --- |
| 2018-05-07 | 0.18 | 0.12 | 10.3 |
| 2018-05-21 | <0.06 | 0.16 | 13.2 |
| 2018-06-04 | 0.64 | 0.72 | 18.1 |
| 2018-06-19 | 0.20 | 1.19 | 17.8 |
| 2018-07-03 | <0.06 | 0.30 | 19.2 |
| 2018-08-29 | 0.16 | 0.54 | 18.8 |
| 2018-09-12 | 0.01 | 0.95 | 17.6 |
| 2018-09-25 | <0.06 | 0.43 | 16.7 |
| 2018-10-09 | <0.06 | 0.19 | 14.1 |
| 2018-10-23 | <0.06 | 0.14 | 13.1 |
| 2018-12-11 | 0.46 | 0.42 | 6.8 |

**Supplementary Table 3.** Summary of the one way ANOVA of growth rates as a function of treatment for each bioassay. Significant differences (p-value< 0.05) are indicated by values in bold.

|  | PE-rich *Synechococcus* | | | PC-rich picocyanobacteria | | | PPE | | |
| --- | --- | --- | --- | --- | --- | --- | --- | --- | --- |
| Date | Df | F value | p-value | Df | F value | p-value | Df | F value | p-value |
| 2018-05-07 | **5** | **29.37** | **<0.001** | **5** | **30.41** | **<0.001** | **5** | **5.59** | **0.006** |
| 2018-05-22 | **5** | **46.77** | **<0.001** | 5 | 2.93 | 0.058 | **5** | **15.57** | **<0.001** |
| 2018-06-04 | 5 | **20.51** | **<0.001** | 5 | 4.89 | 0.113 | **5** | **9.55** | **<0.001** |
| 2018-06-19 | 5 | **7.62** | **0.001** | **5** | **5.85** | **0.005** | **5** | **8.38** | **0.001** |
| 2018-07-03 | 5 | **28.29** | **<0.001** | **5** | **31.95** | **<0.001** | **5** | **23.00** | **<0.001** |
| 2018-08-29 | 5 | 2.31 | 0.108 | 5 | 1.59 | 0.234 | **5** | **4.09** | **0.021** |
| 2018-09-12 | 5 | 2.66 | 0.076 | 5 | 0.97 | 0.076 | 5 | 1.97 | 0.154 |
| 2018-09-25 | 5 | **7.50** | **0.002** | 5 | 2.81 | 0.065 | **5** | **16.78** | **<0.001** |
| 2018-10-09 | 5 | **4.17** | **0.019** | **5** | **30.99** | **<0.001** | **5** | **3.39** | **0.038** |
| 2018-10-23 | 5 | 3.03 | 0.053 | **5** | **4.47** | **0.015** | 5 | 2.32 | 0.107 |
| 2018-12-11 | 5 | 1.34 | 0.311 | 5 | 1.08 | 0.416 | 5 | 3.11 | 0.04 |

**Supplementary Table S4.** P-values of the HSD Tukey comparing PE-rich *Synechococcus* growth rates between treatments of bioassays with significant p-values in Supplementary Table S3. Significant differences (p-value< 0.05) are indicated by values in bold.

|  | 2018-05-07 | 2018-05-22 | 2018-06-04 | 2018-06-19 | 2018-07-03 | 2018-09-25 | 2018-10-09 |
| --- | --- | --- | --- | --- | --- | --- | --- |
| NH_4_- NH_4_+PO_4_ | **<0.001** | **<0.001** | 0.881 | 0.925 | 0.997 | **0.017** | 0.298 |
| NH_4_-NO_3_ | **0.002** | **<0.001** | **0.032** | **<0.001** | **<0.001** | **<0.001** | 0.191 |
| NH_4_-Control | 0.240 | **<0.001** | **<0.001** | 0.999 | **<0.001** | **0.016** | 0.859 |
| NH_4_-NO_3_+PO_4_ | **<0.001** | **<0.001** | **<0.001** | 0.414 | **<0.001** | **0.027** | 0.999 |
| NH_4_-PO_4_ | 0.053 | **<0.001** | **0.002** | 0.191 | **<0.001** | **0.030** | 0.999 |
| NH_4_+PO_4_-NO_3_ | 0.789 | 0.477 | 0.005 | **<0.001** | **<0.001** | 0.417 | 0.999 |
| NH_4_+PO_4_-Control | **<0.001** | **0.026** | **<0.001** | 0.808 | **<0.001** | 1 | 0.050 |
| NH_4_+PO_4_- NO_3_+PO_4_ | 0.906 | **<0.001** | **<0.001** | 0.104 | **<0.001** | 0.999 | 0.462 |
| NH_4_+PO_4_- PO_4_ | 0.074 | **<0.001** | **<0.001** | **0.041** | **<0.001** | 0.999 | 0.211 |
| NO_3_-Control | **<0.001** | 0.452 | 0.118 | **0.015** | 0.997 | 0.437 | **0.030** |
| NO_3_- NO_3_+PO_4_ | 0.269 | **0.017** | 0.201 | 0.228 | 0.999 | 0.290 | 0.314 |
| NO_3_-PO_4_ | 0.480 | **0.013** | 0.655 | 0.477 | 0.971 | 0.271 | 0.132 |
| Control-NO_3_+PO_4_ | **<0.001** | 0.352 | 0.999 | 0.574 | 0.991 | 0.999 | 0.682 |
| Control- PO_4_ | **0.001** | 0.284 | 0.776 | 0.293 | 0.843 | 0.998 | 0.941 |
| PO_4_- NO_3_+PO_4_ | **0.013** | 0.999 | 0.921 | 0.991 | 0.989 | 0.999 | 0.990 |

**Supplementary Table S5.** P-values of the HSD Tukey comparing PC-rich picocyanobacteria growth rates between treatments of bioassays with significant p-values in Supplementary Table S3. Significant differences (p-value< 0.05) are indicated by values in bold.

|  | 2018-05-07 | 2018-06-04 | 2018-06-19 | 2018-07-03 | 2018-10-09 | 2018-10-23 |
| --- | --- | --- | --- | --- | --- | --- |
| NH_4_- NH_4_+PO_4_ | 0.519 | 0.623 | 0.996 | 0.176 | 0.985 | 0.683 |
| NH_4_-NO_3_ | **<0.001** | 0.345 | **0.020** | **<0.001** | 0.694 | 0.571 |
| NH_4_-Control | **<0.001** | 0.159 | 0.979 | **<0.001** | **<0.001** | 0.252 |
| NH_4_-NO_3_+PO_4_ | **<0.001** | 0.502 | 0.120 | **<0.001** | 0.665 | 0.947 |
| NH_4_-PO_4_ | **<0.001** | 0.916 | 0.318 | **0.001** | **<0.001** | 0.849 |
| NH_4_+PO_4_-NO_3_ | **<0.001** | **0.028** | **0.009** | **<0.001** | 0.348 | 0.999 |
| NH_4_+PO_4_-Control | **<0.001** | **0.011** | 0.853 | **<0.001** | **<0.001** | **0.023** |
| NH_4_+PO_4_- NO_3_+PO_4_ | **<0.001** | **0.048** | 0.057 | **<0.001** | 0.325 | 0.988 |
| NH_4_+PO_4_- PO_4_ | 0.346 | 0.181 | 0.165 | **<0.001** | **0.002** | 0.999 |
| NO_3_-Control | 0.962 | 0.993 | 0.064 | 0.786 | **<0.001** | **0.016** |
| NO_3_- NO_3_+PO_4_ | **0.012** | 0.999 | 0.883 | 0.887 | 0.999 | 0.960 |
| NO_3_-PO_4_ | 0.169 | 0.860 | 0.542 | 0.628 | **<0.001** | 0.994 |
| Control-NO_3_+PO_4_ | **0.048** | 0.951 | 0.334 | 0.999 | **<0.001** | 0.064 |
| Control- PO_4_ | **0.046** | 0.574 | 0.683 | 0.999 | 0.516 | 0.040 |
| PO_4_- NO_3_+PO_4_ | **<0.001** | 0.958 | 0.983 | 0.994 | **<0.001** | 0.999 |

**Supplementary Table S6.** P-values of the HSD Tukey comparing PPE growth rates between treatments of bioassays with significant p-values in Supplementary Table S3. Significant differences (p-value< 0.05) are indicated by values in bold.

|  | 2018-05-07 | 2018-05-22 | 2018-06-04 | 2018-06-19 | 2018-07-03 | 2018-08-29 | 2018-09-25 | 2018-10-09 | 2018-12-11 |
| --- | --- | --- | --- | --- | --- | --- | --- | --- | --- |
| NH_4_- NH_4_+PO_4_ | 0.952 | 0.999 | 0.988 | 0.999 | 0.110 | 0.999 | 0.133 | 0.999 | 0.998 |
| NH_4_-NO_3_ | 0.481 | **0.004** | **0.002** | **0.033** | 0.962 | 0.304 | 0.189 | 0.414 | 0.375 |
| NH_4_-Control | 0.289 | **0.005** | 0.999 | 0.313 | **0.002** | **0.022** | 0.953 | 0.999 | 0.999 |
| NH_4_-NO_3_+PO_4_ | 0.175 | **<0.001** | 0.895 | 0.244 | 0.999 | 0.934 | 0.100 | 0.697 | 0.067 |
| N_4_-PO_4_ | 0.459 | **<0.001** | 0.996 | 0.584 | **<0.001** | 0.674 | **0.009** | 0.516 | 0.770 |
| NH_4_+PO_4_-NO_3_ | 0.913 | **0.006** | **<0.001** | **0.036** | 0.029 | 0.384 | **0.002** | 0.532 | 0.569 |
| NH_4_+PO_4_-Control | 0.732 | **0.007** | 0.999 | 0.288 | **<0.001** | **0.030** | **0.033** | 0.991 | 0.999 |
| NH_4_+PO_4_- NO_3_+PO_4_ | 0.536 | **<0.001** | 0.588 | 0.267 | 0.184 | 0.972 | **0.001** | 0.571 | 0.122 |
| NH_4_+PO_4_- PO_4_ | 0.141 | **<0.001** | 0.887 | 0.619 | **<0.001** | 0.772 | 0.632 | 0.642 | 0.924 |
| NO_3_-Control | 0.998 | 0.999 | **0.001** | **<0.001** | **0.007** | 0.594 | 0.560 | 0.259 | 0.472 |
| NO_3_- NO_3_+PO_4_ | 0.971 | 0.768 | **0.010** | 0.808 | 0.862 | 0.789 | 0.998 | **0.045** | 0.857 |
| NO_3_-PO_4_ | **0.027** | 0.839 | **0.004** | 0.420 | **0.003** | 0.977 | **<0.001** | 0.999 | 0.975 |
| Control-NO_3_+PO_4_ | 0.999 | 0.722 | 0.784 | **0.006** | **0.001** | 0.104 | 0.350 | 0.872 | 0.092 |
| Control- PO_4_ | **0.014** | 0.799 | 0.977 | **0.022** | 0.996 | 0.251 | **0.002** | 0.338 | 0.861 |
| PO_4_- NO_3_+PO_4_ | **0.007** | 0.999 | 0.990 | 0.977 | **<0.001** | 0.990 | **<0.001** | 0.062 | 0.468 |

# References

Bertilsson, S., Berglund, O., Karl, D. M., & Chisholm, S. W. (2003). Elemental composition of marine *Prochlorococcus* and *Synechococcus*: Implications for the ecological stoichiometry of the sea. *Limnol. Oceanogr.*, *48*(5), 1721–1731. https://doi.org/10.4319/lo.2003.48.5.1721

Campbell, L., Nolla, H. A., & Vaulot, D. (1994). The importance of *Prochlorococcus* to community structure in the central North Pacific Ocean. *Limnol. Oceanogr.*, *39*(4), 954–961. https://doi.org/10.4319/lo.1994.39.4.0954

Cuhel, R. L., & Waterbury, J. B. (1984). Biochemical composition and short term nutrient incorporation patterns in a unicellular marine cyanobacterium, *Synechococcus* (WH7803). *Limnol. Oceanogr.*, *29*(2), 370–374. https://doi.org/10.4319/lo.1984.29.2.0370

Durand, M. D., Olson, R. J., & Chisholm, S. W. (2001). Phytoplankton population dynamics at the Bermuda Atlantic Time-series station in the Sargasso Sea. *Deep Sea Res. Part II Top. Stud. Oceanogr.*, *48*(8–9), 1983–2003. https://doi.org/10.1016/S0967-0645(00)00166-1

Edler, L. (1979). *Recommendations on methods for marine biological studies in the Baltic Sea. Phytoplankton and chlorophyll*. Baltic Marine Biologists BMB (Sweden).

Fu, F.-X., Warner, M. E., Zhang, Y., Feng, Y., & Hutchins, D. A. (2007). Effects of increased temprature and CO2 on photosynthesis, growth and elemental ratios in marine *Synechococcus* and *Prochlorococcus* (cyanobacteria). *Eur. J. Phycol.*, *43*(3), 485–496. https://doi.org/10.1111/j.1529-8817.2007.00355.x

Kana, T. M., & Glibert, P. M. (1987). Effect of irradiances up to 2000 μE m^-2^ s^-1^ on marine *Synechococcus* WH7803-I. Growth, pigmentation, and cell composition. *Deep Sea Res. I: Oceanogr. Res. Pap.*, *34*(4), 479–495. https://doi.org/10.1016/0198-0149(87)90001-X

Liu, H., Bidigare, R. R., Laws, E., Landry, M. R., & Campbell, L. (1999). Cell cycle and physiological characteristics of *Synechococcus* (WH7803) in chemostat culture. *Mar. Ecol. Prog. Ser.*, *189*(1988), 17–25. https://doi.org/10.3354/meps189017

Liu, H., Chang, J., Tseng, C. M., Wen, L. S., & Liu, K. K. (2007). Seasonal variability of picoplankton in the Northern South China Sea at the SEATS station. *Deep Sea Res. Part II Top. Stud. Oceanogr.*, *54*(14–15), 1602–1616. https://doi.org/10.1016/j.dsr2.2007.05.004

Llewellyn, C. A., & Gibb, S. W. (2000). Intra-class variability in the carbon, pigment and biomineral content of prymnesiophytes and diatoms. *Mar. Ecol. Prog. Ser.*, *193*, 33–44. https://doi.org/10.3354/meps193033

Montagnes, D. J. S., John, A. B., Paul J., H., & Taylor, F. J. R. (1994). Estimating carbon , nitrogen , protein , and chlorophyll a from volume in marine phytoplankton. *Limnol. Oceanogr.*, *39*(5), 1044–1060.

Paczkowska, J., Brugel, S., Rowe, O., Lefébure, R., Brutemark, A., & Andersson, A. (2020). Response of coastal phytoplankton to high inflows of terrestrial matter. *Front. Mar. Sci.*, *7*, 80. https://doi.org/10.3389/fmars.2020.00080

Sondergaard, M., Jensen, L. M., & Aertebjerg, G. (1991). Picoalgae in Danish coastal waters during summer stratification. *Mar. Ecol. Prog. Ser.*, *79*(1–2), 139–149. https://doi.org/10.3354/meps079139

Valderrama, J. C. (1995). Methods of nutrient analysis. In G. M. Hallagraeff, D. M. Anderson, & A. D. Cembella (Eds.), Manual on Harmful Marine Microalgae (pp. 251–268). IOC Manuals and Guides.

Veldhuis, M. J. W., Kraay, G. W., Van Bleijswijk, J. D. L., & Baars, M. A. (1997). Seasonal and spatial variability in phytoplankton biomass, productivity and growth in the northwestern Indian ocean: The southwest and northeast monsoon, 1992-1993. *Deep Sea Res. I: Oceanogr. Res. Pap.*, *44*(3), 425–449. https://doi.org/10.1016/S0967-0637(96)00116-1

Verity, P. G., Robertson, C. Y., Tronzo, C. R., Andrews, M. G., Nelson, J. R., & Sieracki, M. E. (1992). Relationships between cell volume and the carbon and nitrogen content of marine photosynthetic nanoplankton. *Limnol. Oceanogr.*, *37*(7), 1434–1446. https://doi.org/10.4319/lo.1992.37.7.1434

Wei, Y., Huang, D., Zhang, G., Zhao, Y., & Sun, J. (2020). Biogeographic variations of picophytoplankton in three contrasting seas: The Bay of Bengal, South China Sea and western Pacific Ocean. *Aquat. Microb. Ecol.*, *84*(1), 91–103. https://doi.org/10.3354/ame01928

Wei, Y., Sun, J., Zhang, X., Wang, J., & Huang, K. (2019). Picophytoplankton size and biomass around equatorial eastern Indian Ocean. *Microbiologyopen*, *8*(2), e629. https://doi.org/10.1002/mbo3.629

Worden, A. Z., Nolan, J. K., & Palenik, B. (2004). Assessing the dynamics and ecology of marine picophytoplankton: The importance of the eukaryotic component. *Limnol. Oceanogr.*, *49*(1), 168–179. https://doi.org/10.4319/lo.2004.49.1.0168

Zubkov, M. V., Sleigh, M. A., Tarran, G. A., Burkill, P. H., & Leakey, R. J. G. (1998). Picoplanktonic community structure on an Atlantic transect from 50°N to 50°S. *Deep Sea Res. I: Oceanogr. Res. Pap.*, *45*(8), 1339–1355. https://doi.org/10.1016/S0967-0637(98)00015-6
